# Conformational heterogeneity in V1/V2 domain affects the immunological properties of this region

**DOI:** 10.1186/1742-4690-9-S2-P307

**Published:** 2012-09-13

**Authors:** A Pinter, AZ Wu, T Neubert, MD Burkhart, WJ Honnen

**Affiliations:** 1 University of Medicine and Dentistry of New Jersey, Newark, NJ, USA; 2 Nanjing University, Nanjing, China; 3 NYU Medical Center, New York, NY, USA

## Background

Recent information has shown the importance of the V1/V2 domain as a target in HIV-1 vaccines. This includes the localization of epitopes to potently neutralizing antibodies to this region, the demonstration that binding between a site in V1/V2 and α4β7 receptors facilitates infection in primary CD4-positive T cells, and the demonstration that the presence of antibodies binding to the native form of the V1/V2 domain of HIV-1 gp120 correlated with protection in the RV144 vaccine trial. These results highlight the need to better understand the structure and immunological properties of this region, in order to optimally express the relevant targets in HIV-1 vaccines.

## Methods

The native V1/V2 domain of a clade B sequence (CaseA2) was expressed by fusion to the C-terminus of a 273 aa fragment of the MuLV gp70 domain. This fusion glycoprotein was characterized by SDS-PAGE under various conditions, by radioimmunoprecipitation experiments with a panel of mAbs directed against V1/V2-specific epitopes, and by MALDI-TOF analysis of immunologically fractionated forms.

## Results

SDS-PAGE analysis of this protein after deglycosylation under non-reducing conditions revealed the presence of a closely migrating doublet, and mass-spec analysis of the immunologically fractionated forms suggested that these consisted of different disulfide-linked conformers. The two forms reacted differentially with a panel of V1/V2-specific antibodies and possessed variable reactivity with different polyclonal sera.

## Conclusion

These studies identify a conformational heterogeneity in the V1/V2 domain that is due to alternative disulfide bonding patterns. These conformations profoundly affect the immunoreactivity of this region, and will presumably influence their immunogenicity as well. Due to the importance of this region as a target for neutralizing and otherwise protective antibodies, it is likely that methods for resolving this heterogeneity would improve the efficacy of induction of relevant antibodies to this region by HIV Env vaccines.

